# Comparison of Changes in the Condylar Volume and Morphology in Skeletal Class III Deformities Undergoing Orthognathic Surgery Using a Customized versus Conventional Miniplate: A Retrospective Analysis

**DOI:** 10.3390/jcm9092794

**Published:** 2020-08-30

**Authors:** You Na Lim, In-Young Park, Jong-Cheol Kim, Soo-Hwan Byun, Byoung-Eun Yang

**Affiliations:** 1 Division of Orthodontics, Hallym University Sacred Heart Hospital, Anyang 14066, Korea; 1004lun@hanmail.net (Y.N.L.); denti2875@hallym.or.kr (I.-Y.P.); 2 Graduate School of Clinical Dentistry, Hallym University, Chuncheon 24252, Korea; ddskjc@hanmail.net (J.-C.K.); purheit@daum.net (S.-H.B.); 3 Institute of Clinical Dentistry, Hallym University, Chuncheon 24252, Korea; 4 Mir Dental Hospital, Daegu 41940, Korea; 5 Division of Oral and Maxillofacial Surgery, Hallym University Sacred Heart Hospital, Anyang 14066, Korea

**Keywords:** bone plate, condylar position, orthognathic surgery, CAD/CAM, digital surgery

## Abstract

With the great leap in the development of three-dimensional computer-assisted surgical technology, surgeons can use a variety of assistive methods to achieve better results and evaluate surgical outcomes in detail. This retrospective study aimed to evaluate the postoperative stability after bilateral sagittal split ramus osteotomy by volume rendering methods and to evaluate how postoperative stability differs depending on the type of surgical plate. Of the patients who underwent BSSRO, ten patients in each group (non-customized miniplate and customized miniplate) who met the inclusion criteria were selected. Preoperative and postoperative cone-beam computed tomography data were collected, and condylar morphological and landmark measurements were obtained using Checkpoint and OnDemand software, respectively. The postoperative condylar morphological dataset revealed no significant difference (*p* > 0.05) between the two groups. No significant difference (*p* > 0.05) was observed between the two groups in horizontal, vertical, or angular landmark measurements used to quantify operational stability. These results indicate that there is no difference in the surgical outcome between the patient-specific system and the conventional method, which will allow clinicians to take advantage of the patient-specific system for this surgical procedure, with favorable results, as with the conventional method.

## 1. Introduction

A severe skeletal discrepancy in adults with complete growth can only be treated with orthognathic surgery (OGS). OGS has yielded better outcomes through improved surgical planning and procedures [1]. However, OGS can lead to changes in the healthy relationship of the jaw bones, and these changes may affect the postoperative stability and abnormalities of the temporomandibular joint (TMJ) [2,3,4].

One of the factors involved in relapse is the type of fixation method applied for bone fragments, and adjustment of the proximal segment, including the mandibular condyle, is the most crucial factor affecting skeletal stability and relapse after OGS [3,4]. Postoperative relapse is affected by changes in the position and shape of the condyle, as well as the movement of the bone fragments at the osteotomy site [5]. A previous study has shown that skeletal relapse occurring less than six months after surgery is often associated with inadequate unilateral or bilateral condylar positioning, causing sagging of the condyle and resulting in an undesirable displacement of the mandible [6].

Many scholars have also found that changes in the position of the mandible can cause or worsen TMJ disorders and affect the stability of the mandible. Furthermore, various methods have been introduced to maintain the position of the mandibular condyle [5,7,8,9].

The advancement of three-dimensional (3D) computer technology provides the basis of a paradigm shift in surgical procedures and outcomes. Computer-aided design and computer-aided manufacturing (CAD/CAM) technologies enable accurate surgical planning specifically by controlling the position of the condyle [10]. In our previous study, we reported the accuracy and stability of OGS results with a newly developed computer-aided surgical simulation (CASS) method [11].

Additionally, various 3D imaging programs help clinicians predict the operative results and visualize postoperative skeletal and condylar morphological changes. Three-dimensional-based volumetric methods have recently been utilized to quantify postoperative changes to overcome the shortcomings of linear measurements [12].

The purpose of this study was to evaluate the postoperative stability of the condylar position after bilateral sagittal split ramus osteotomy (BSSRO) by volume rendering methods and to evaluate how postoperative stability differs depending on the type of surgical plate. 

We hypothesized that the use of a customized miniplate in OGS would not be better in the surgical results concerning the TMJ change.

## 2. Materials and Methods

### 2.1. Study Design and Sample

This retrospective study involved the medical records of patients who had undergone BSSRO from January 2012 to December 2018 at Hallym University Hospital. The included study samples fulfilled the following criteria: (1) skeletal Class III malocclusion (ANB < 0°) treated with BSSRO with or without Le Fort I and genioplasty; (2) no complications such as temporomandibular disorder, reoperation, and fixation failure after surgery; and (3) availability of cone-beam computed tomography (CBCT) data obtained at initial and follow-up examinations.

Patients were excluded from this study if (1) there was a pathological condition affecting the condyles or maxillofacial bones (e.g., osteochondroma, congenital genetic deformities) or (2) imaging data were either absent or insufficient.

**Figure 1:**
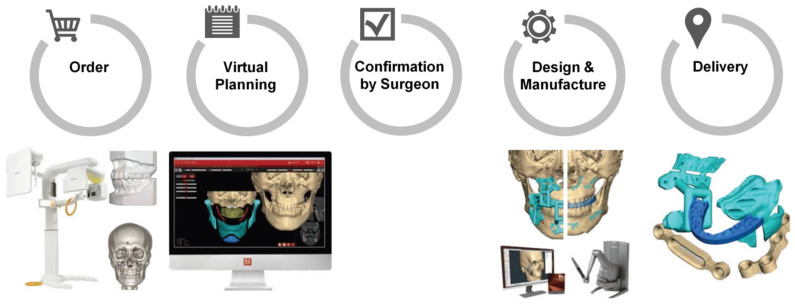
Work process of orthognathic surgery with the computer-aided surgical simulation (CASS) system using FaceGide^®^ [11].

According to the type of miniplate, the study samples were divided into the non-customized miniplate group (NCP group) and the customized miniplate group (CP group). In the CP group, the patients underwent OGS with the CASS system using FaceGide^®^ (MegaGen Co., Daegu, Korea) from March 2015, consisting of computer-aided surgical planning and design and the use of 3D-printed cutting guides and plates for BSSRO (Figure 1). This study was approved by the institutional review board of Hallym University Sacred Heart Hospital (approval No. 2020-07-035).

### 2.2. Treatment Protocols and CBCT Imaging

**Figure 2:**
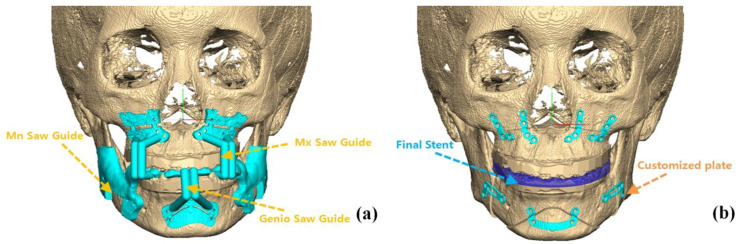
Virtual 3D skeletal images merged on scanned dental casts: (**a**) cutting guides for Le-Fort I osteotomy, BSSRO, and genioplasty, (**b**) dental arches were fit into the final splint, and customized plates were placed on repositioned bone fragments.

**Figure 3:**
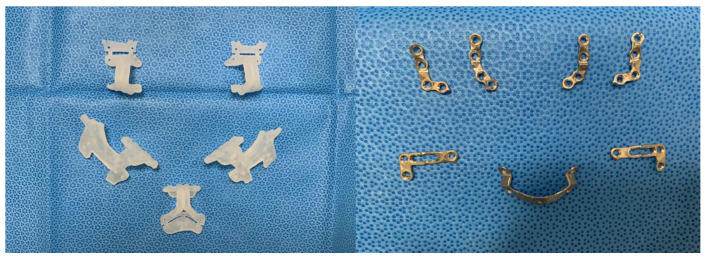
Actual 3D-printed cutting guides (**left**) and miniplates (**right**). The predicted drilling holes are placed on the guides with the osteotomy line marked. The plate printed according to the bony contour does not require bending.

After preoperative orthodontic treatment, all patients underwent surgery by one surgeon (B.E.Y). In the NCP group, surgical planning was operated with a CASS system (InVivoDental v5.0 (Anatomage, San Jose, CA, USA)); however, titanium plates were bent and applied directly in the operation room to secure the moved bone fragments. In OGS of the NCP group, condyle positioning was performed through a method developed by our institution [13]. In the CP group, plates were premade according to the preoperative 3D diagnosis and design (Figure 2 and Figure 3). During OGS in the CP group, condyle positioning was performed using a customized plate and proximal segment positioning device [14], not the method used in the NCP group.

Intermaxillary fixation was performed using surgical archwires after surgery to stabilize the occlusion. After one week, the surgical splint was removed prior to CBCT taking, and the patients initiated postoperative orthodontic treatment a month later. Postoperative orthodontic treatment was performed to obtain stable occlusion, proper tooth alignment, dental midline correction, and an appropriate overjet and overbite. The duration of postoperative orthodontic treatment was on average 9 months for the NCP group and 10.2 months for the CP group.

CBCT images were obtained three times: preoperatively (T0), at one week postoperatively (T1), and at four months postoperatively (T2) using CBCT (Alphard 3030; Asahi Inc., Kyoto, Japan). All images were obtained in centric occlusion at 80 kVp, 5 mA, and an exposure time of 17 s and were transformed into the DICOM format.

### 2.3. Condylar Volume Rendering

**Figure 4:**
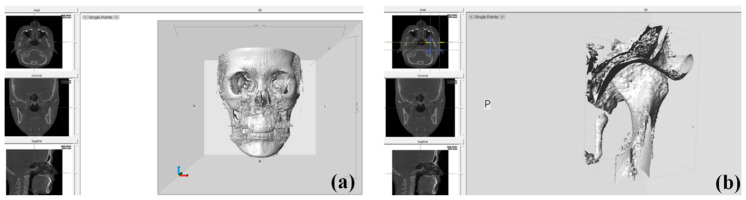
The view of Checkpoint (Stratovan): (**a**) imported CBCT data that were exported in DICOM format, (**b**) cropped volume isolating the left condyle-fossa unit.

DICOM images obtained at T0 and T2 were reconstructed three-dimensionally using Checkpoint software (Stratovan, Davis, CA, USA), a new semiautomatic landmark software program for 3D analysis of the relationship among the osseous components of the TMJ. Condyle-fossa units were isolated from DICOM data using a cropping tool and subsequently exported to 3D images (Figure 4). 

**Figure 5:**
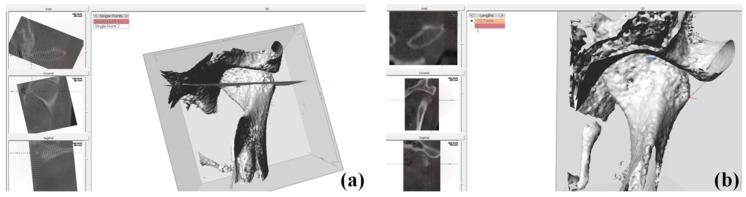
Linear measurements of condyle on Checkpoint: (**a**) single points placed on the medial and lateral pole of the condylar head, (**b**) distance measurement between the medial and lateral pole of the condylar head (diameter).

The linear measurements, such as the height, width, and diameter of the cropped condylar head, were measured in the 3D view (Table 1, Figure 5). The volumetric measurements, such as the condylar head volume and joint space volume, were obtained through several points and landmarks.

**Figure 6:**
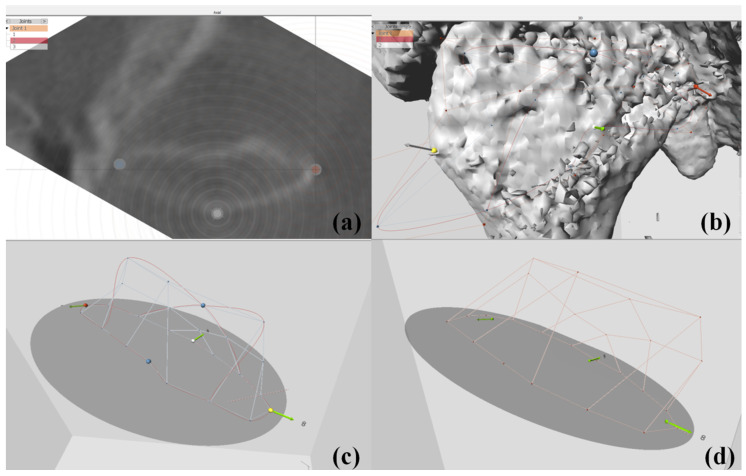
Volumetric measurement of the condyle-fossa unit on Checkpoint: (**a**) axial view of three anchor points placed in the medial, lateral, and posterior pole of the condylar head, (**b**) three-dimensional view of three anchor points displayed in yellow, red, and white dots, (**c**) the shape of the condylar head obtained via a 5 × 5 semi-automated landmark with three anchor points, (**d**) the shape of the joint space obtained via a 5 × 5 semi-automated landmark with three anchor points.

Anchor points were digitized on the condylar head and together served as an equator around the condyle-fossa unit to create a semi-landmark patch (Table 1, Figure 6). Semi-landmarks with a 5 × 5 patch density (number of points around a given area) were automatically placed over the condyle-fossa surface (Figure 6).

### 2.4. Evaluation of Skeletal Landmark Changes after Surgery 

**Figure 7:**
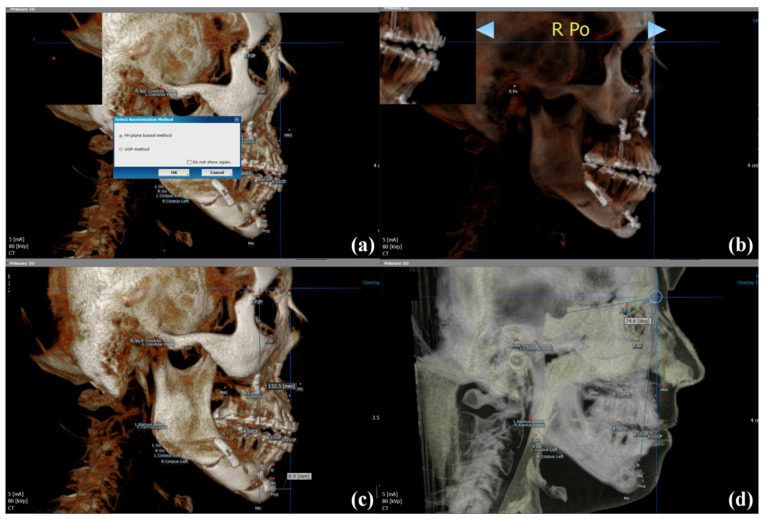
Landmark measurements for skeletal stability on OnDemand (Cybermed): (**a**) selection of orientation method, (**b**) image orientation along the Frankfurt Horizontal (FH) plane based methods on the basis of the right porion, right orbitale, and left orbitale, (**c**) horizontal and vertical measurements on 3D skeletal view; horizontal reference plane (HRP) to menton and vertical reference plane (VRP) to pogonion, respectively, (**d**) angular measurements on adjusted opacity value; SN to NB.

DICOM images obtained at T1 and T2 were reconstructed three-dimensionally using OnDemand 3D software (Cybermed, Seoul, Korea). Images were reoriented along the Frankfort horizontal plane in reference to the right porion, right orbitale, and left orbitale. Then, the orthogonal reference planes were set accordingly, that is, the sagittal plane (right to left cross-sections), axial or horizontal plane (top to bottom cross-sections), and coronal or frontal plane (front to back cross-sections). The relation between the vertical reference plane (VRP) and pogonion (Pog), the horizontal reference plane (HRP) and menton (Me), and the Sella-Nasion (SN) and Nasion-B point (NB) (SNB) was measured in 3D cephalometric mode to quantify skeletal changes from T1 to T2 (Table 2, Figure 7). All measurements and software operations were performed by a single examiner (LYN)

### 2.5. Statistical Analysis

Statistical analysis was performed using SPSS version 16.0 (IBM Co., Armonk, NY, USA). The normal distribution of measurements was verified by the Shapiro–Wilk test. The independent *t*-test was used to assess differences in mean condylar morphological measurements at T0 and T2 and skeletal changes between the NCP and CP groups. The paired *t*-test was applied to evaluate condylar morphological changes (T0 to T2) and post-surgical stability (T1 to T2) in the two groups. Differences were considered statistically significant at *p* < 0.05.

## 3. Results

Among the patients who underwent OGS, twenty-two patients (from 2012 to 2015) with conventional (ready-made) miniplates and twenty-five patients (from 2015 to 2018) with customized miniplates were investigated. On the basis of the inclusion and exclusion criteria, cases were excluded mostly due to insufficient data, twenty patients (11 males and 9 females; mean age, 25 years) were finally selected. Of these, ten patients were included in the NCP group (five males and five females; mean age, 25.2 years), and ten patients were included in the CP group (six males and four females; mean age, 24.8 years) (Table 3). 

### 3.1. Condylar Morphological Changes (T0 vs.T2)

Significant differences in the left and right condylar head morphology and joint space volume were verified with the *t*-test. For all but the left and right joint space volumes in the NCP group, there were no significant differences in the values on the left and right sides of the condyle in either group (Table 4). The changes in the condylar head morphology and condylar joint space volume in the two groups before surgery (T0) and four months after surgery (T2) are described in Table 5. No significant difference was evident between the two groups regarding condylar morphological changes from T0 to T2, except for the left condylar height.

### 3.2. Skeletal Landmark Changes (T1 vs. T2)

Table 6 shows the skeletal changes from T1 to T2, in which the VRP to Pog distance represents horizontal changes, the HRP to Me distance represents vertical changes, and the SNB represents angular changes. Forward movement within 1 mm on average occurred in both groups (NCP; 0.46 mm, CP; 0.75 mm), and upward movement occurred 0.61 mm in the NCP group and 1.08 mm in the CP group. The SNB angle increased less than 1 degree in both groups (NCP; 0.68°, CP; 0.47°). The vertical, horizontal, and angular changes in the two groups from T1 to T2, however, were not significantly different. There were also no significant differences in the skeletal changes (horizontal, vertical, angular) between the two groups.

## 4. Discussion

The surgical method and procedure determine the stability after repositioning of the jaw bones. This retrospective study compared postoperative skeletal changes between the NCP and CP groups. We focused on changes in the condylar head and joint space among the many factors affecting relapse after OGS. Wan et al. reported the effect of virtual surgical planning (VSP) on the accuracy of condylar seating in surgical cases related to BSSRO and assessed differences between the actual surgical results and planned virtual results. They concluded that VSP did not lessen changes in the condylar position or angulation compared with conventional planning in OGS, which is similar to our findings [15]. 

Since true relapse only occurs in some cases, and some significant values can contribute to the results, statistical measurements can be misleading. It is therefore better to consider the proportion of patients with clinically significant changes, rather than the mean values of the differences [16]. In a study by da Silva et al., after OGS, 54.4% of the condyles showed changes in volume; in other words, the volume change was at least 10% of the original volume. A decrease in volume was found in 33.3% of the condyles, while an increase in volume was observed in 21.1% of the condyles [7]. In our study, the condylar head volume changed in 67.5% of condyles, increasing in 40.5% of condyles and decreasing in 27% of condyles at T2. Even though endochondral growth is expected to occur in adulthood, these results may indicate that after the condyle has been repositioned, it is able to generate new bone via an adaptive biomechanical process [17]. There was no correlation between change in the volume and joint space. Therefore, the factor other than condylar remodeling, such as condylar displacement, is more likely related to changes in the joint spaces [7]. 

Postoperative skeletal relapse in patients with skeletal Class III malocclusion is defined as an anterior movement of the mandible after OGS [18,19]. In this study, most skeletal changes indicated forward and upward movement for approximately four months after OGS. Proffit et al. reported that approximately 50% of postoperative skeletal relapses occurred in the first six weeks immediately after functional reactivation [20]. Stable occlusal contacts can make the postoperative outcome more predictable. Since occlusal settling through post-surgical orthodontic treatment allows eliminating the posterior occlusal interferences, the mandible rotated counterclockwise, providing vertical and horizontal movement to compare to the immediate postoperative position [21]. 

Surgical accuracy can be significantly improved using CAD/CAM techniques [22]. Most authors agree that these new systems provide an excellent level of accuracy in OGS that is generally higher than that obtained using classic methods [8,10,22,23,24]. Nevertheless, the quality of the surgical results is still determined by the individual surgeon’s skill in executing the surgical plan. However, many studies have shown that clinicians can increase surgical safety, shorten the operative duration, and improve the predictability of the surgical results using patient-specific systems based on 3D technology [22,23,24]. Recently, 3D color mapping has enabled 3D visualization of the skeletal changes to the condyle after surgical manipulation of the jaw bones, and this technique can be applied to monitor positional changes of the condyle. This method provides a general view of changes, but it cannot evaluate 3D changes as a more depictive scale [25,26,27]. Schilling et al. found landmark-based and voxel-based techniques to be convincing and helpful to quantify delicate differences in the 3D condylar morphology [28]. Ikeda et al. used reliable anchor points that could be used to generate a reproducible equator at the height of the medial and lateral poles to enable semi-automated landmark estimation. This new semi-automatic method is a reliable tool for the 3D analysis of the form of the condylar head and joint space [29]. 

The number of semi-landmarks, and the patch density, can be selected in the Stratovan Checkpoint software. The semi-landmarks are placed over the structure of interest to capture their shape. As the patch density increased from 5 × 5 to 13 × 13, the number of landmarks increased, and more detailed contours of the condyle were visually captured. The 5 × 5 and 7 × 7 patches often failed to capture concavities on the condylar surface, and the 9 × 9 patches did not quite accurately capture the defect. The 11 × 11 patch provided subtle changes in the condylar head with much more detail [27]. Nonetheless, our research proceeded with a patch density of 5 × 5. We are aware that more massive datasets would provide more detailed information regarding shape; however, the large number of landmarks to observe is beyond the number of landmarks that are manageable by one examiner.

Cevidanes et al. concluded that condylar displacement was not significant in two-jaw surgery compared with maxilla-only surgery [30]. On the other hand, Kim et al. evaluated the condylar positional changes after one-jaw and two-jaw correction for mandibular prognathism and verified significant angular changes in the condyle after surgery in the two-jaw group. They postulated that the surgical technique could play an essential role in passive condylar seating [9]. Genioplasty also can be an influential factor in the results of this study. The short- and long-term stability was evaluated since the prolongation of the soft tissue, and the direction of the suprahyoid muscles can be changed according to the bony movement. However, it was concluded to be stable without any specific skeletal relapse [16]. We suppose that there would be no significant effect on the results as BSSRO-only and two-jaw surgery patients were similarly distributed in the two groups in our study, and genioplasty would not have a significant impact on the outcome as well.

The time of CBCT taking at T2 might be questionable because it seemed to be quite short-term to evaluate the changes after OGS. The formation of primary bony callus is complete, and the bone stabilizes approximately four months after surgery [31]. In the study of Miura et al., the authors sonographically assessed bone formation, and the bone gap had disappeared in 6 of 10 cases at four months postoperatively [32].

One of the reasons for the small sample size was the application of strict inclusion and exclusion criteria, and a large number of patients who underwent OGS with a conventional method were excluded because of a lack of imaging data. Despite the small sample size, analysis with volumetric measurements in 3D programs facilitates a more detailed examination of multiple dimensions in a flexible, continuous, and automatic way, which can lead to quite reliable results.

In our study, OGS with a patient-specific system could be entirely consistent with the planned outcome, but changes in the position of the condyle and relapse were inevitable. However, there have been few studies on how the surgical outcomes of patient-specific systems differ from those of conventional methods. In further research, a comparative study of the surgical outcomes of customized and conventional methods can be conducted from various perspectives to complement the limitations of this study.

## 5. Conclusions

The condylar changes in both groups before and after OGS were similar. The rate of short-term postoperative relapse was not significantly different between the NCP and CP groups.

In other words, there was no remarkable difference in the surgical results in terms of the condylar volume and morphology. Even so, the patient-specific system is highly predictable, efficient, and saves surgical time; thus, the utilization of this system in OGS should be considered.

## Figures and Tables

**Table 1:** Definition of condylar morphologic landmarks and measurements.

Anchor Points	Description
Medial point (red)	A most prominent point on the medial contour of the mandibular condyle
Lateral point (yellow)	A most prominent point on the lateral contour of the mandibular condyle
Posterior point (white)	A most prominent point on the posterior contour of the condyle where the cortication tapers to an even thickness
Height	Distance between the most superior part of the condylar head to a line through medial and lateral anchor point in coronal slice
Diameter	Distance between the most prominent point on the medial to the lateral side of the mandibular condyle in coronal slice
Width	Distance between the point on the posterior to the anterior contour of the condyle in axial slice
Condylar head volume	The volume of condyle above the plane consisting of anchor points
Joint space volume	The volume of fossa above the plane consisting of anchor points

**Table 2:** Definition of 3D cephalometric landmarks and measurements.

Landmarks	Original Terms	Definitions
S	Sella	Center of the pituitary fossa of the sphenoid bone
N	Nasion	The most anterior point of the nasofrontal suture
B	B point	The point of maximum concavity in the midline of the alveolar process of mandible
Pog	Pogonion	The most anterior point of the symphysis of the mandible
Me	Menton	The most inferior point on the symphysis of the mandible
SNB	SN to NB	The angle between the SN plane and a plane connecting the NB
VRP	Vertical Reference plane	The plane perpendicular to the FH plane passing through the nasion
HRP	Horizontal reference plane	The plane parallel to the FH plane passing through the nasion

**Table 3:** Sample characteristics (*n* = 20).

Variables	Non-Customized (*n* = 10)	Customized (*n* = 10)
Age	25.2 ± 4.66	24.8 ± 3.88
Sex		
Male	5(50%)	6(60%)
Female	5(50%)	4(40%)
Surgery		
Lefort I	6(60%)	6(60%)
Genioplasty	3(30%)	4(40%)
BSSRO	10(100%)	10(100%)

**Table 4:** Mean values of right and left condylar morphology at T0 and T2 for each group.

Time	Non-Customized			Customized		
	Right	Left	*p*-Value ^1^	Right	Left	*p*-Value ^1^
Height (mm)						
T0	7.67 ± 1.43	7.01 ± 1.21	0.279	9.06 ± 1.32	8.68 ± 1.66	0.578
T2	7.25 ± 1.7	6.65 ± 1.43	0.404	9.68 ± 0.87	9.06 ± 1.63	0.302
Diameter (mm)						
T0	20.95 ± 2.13	21 ± 1.45	0.952	21.73 ± 1.97	21.23 ± 2.86	0.185
T2	20.62 ± 2.08	20.96 ± 1.7	0.693	21.49 ± 2.14	21.27 ± 3.1	0.856
Width (mm)						
T0	7.15 ± 0.7	7.46 ± 1.21	0.492	9.65 ± 1.75	8.74 ± 2.63	0.374
T2	7.38 ± 0.66	7.34 ± 1.37	0.935	9.94 ± 1.6	9.04 ± 2.67	0.372
Condylar head volume (mm^3^)						
T0	593.52 ± 174.01	483.55 ± 169.12	0.169	775.83 ± 152.22	664.84 ± 271.17	0.274
T2	578.58 ± 180.55	485.03 ± 191.52	0.276	799.83 ± 181.02	694.47 ± 224.99	0.264
Joint space volume (mm^3^)						
T0	1001.91 ± 304.04	701.22 ± 282.43	<0.05 *	1019.38 ± 264.03	942.91 ± 339.15	0.581
T2	1082.44 ± 474.55	785.17 ± 284.17	0.106	1087.5 ± 159	1022.43 ± 328.15	0.582

* *p* < 0.05. For the definition of parameters, please refer to Table 2. mm, millimeters; mm^3^, cubic millimeters; T0, before surgery; T2, four months after surgery; *p*-values ^1^ by independent *t*-test; values are presented as mean ± standard deviation.

**Table 5:** Comparison of the condylar morphologic changes between the two groups (T2 vs. T0).

Side	Non-Customized		Customized		Between-Group (*p*-Value ^2^)
	Diff.	*p*-Value ^1^	Diff.	*p*-Value ^1^	
Height (mm)					
Right	−0.42 ± 1.11	0.262	0.62 ± 1.31	0.169	0.071
Left	−0.36 ± 0.58	0.083	0.38 ± 0.58	0.067	<0.05 *
Diameter (mm)					
Right	−0.33 ± 0.63	0.133	−0.24 ± 0.69	0.3	0.765
Left	−0.04 ± 0.44	0.779	0.04 ± 0.56	0.827	0.727
Width (mm)					
Right	0.23 ± 0.51	0.188	0.29 ± 1.2	0.463	0.886
Left	−0.12 ± 0.42	0.388	0.3 ± 0.65	0.177	0.102
Condylar head volume (mm^3^)					
Right	−14.94 ± 82.34	0.58	24 ± 114.72	0.525	0.395
Left	1.48 ± 74.98	0.952	29.63 ± 124.01	0.469	0.547
Joint space volume (mm^3^)					
Right	80.53 ± 253.54	0.341	68.12 ± 254.54	0.419	0.914
Left	83.95 ± 124.98	<0.05 ^*^	79.52 ± 206.1	0.253	0.954

* *p* < 0.05. For the definition of parameters, please refer to Table 2. Diff, the difference between T0 and T2; mm, millimeters; mm^3^, cubic millimeters; T0, before surgery; T2, four months after surgery; *p*-value ^1^ by paired *t*-test; *p*-values ^2^ by independent *t*-test; values are presented as mean ± standard deviation. Positive and negative values represent increases and decreases in measurements at T2, respectively.

**Table 6:** Comparison of skeletal changes between the two groups (T2 vs. T1).

Parameters	Non-Customized		Customized		Between-Group (*p*-Value ^2^)
	Diff.	*p*-Value ^1^	Diff.	*p*-Value ^1^	
N-perp to pog (mm) ^¶^	0.46 ± 2.35	0.551	0.75 ± 1.61	0.174	0.751
HRP to Me (mm) ^§^	0.61 ± 1.05	0.099	1.08 ± 1.55	0.055	0.438
SNB (°) ^§§^	0.68 ± 1.44	0.169	0.47 ± 0.8	0.096	0.692

For the definition of landmarks, please refer to Table 3; Diff, the difference between T0 and T2; mm, millimeters; ° degree (angle) T1, one week after surgery; T2, four months after surgery; *p*-value ^1^ by paired *t*-test, *p*-values ^2^ by independent *t*-test; values are presented as mean ± standard deviation. ^¶^ Positive and negative values represent the forward and backward movement of pogonion at T2, respectively. ^§^ Positive and negative values represent the upward and downward movement of menton at T2, respectively. ^§§^ Positive and negative values represent increases and decreases in the angle of SNB at T2, respectively.
